# Measuring and forecasting progress in education: what about early childhood?

**DOI:** 10.1038/s41539-021-00106-7

**Published:** 2021-09-10

**Authors:** Linda M. Richter, Jere R. Behrman, Pia Britto, Claudia Cappa, Caroline Cohrssen, Jorge Cuartas, Bernadette Daelmans, Amanda E. Devercelli, Günther Fink, Sandra Fredman, Jody Heymann, Florencia Lopez Boo, Chunling Lu, Elizabeth Lule, Dana Charles McCoy, Sara N. Naicker, Nirmalo Rao, Abbie Raikes, Alan Stein, Claudia Vazquez, Hirokazu Yoshikawa

**Affiliations:** 1 DSI-NRF Centre of Excellence in Human Development, University of the Witwatersrand, Johannesburg, South Africa; 2 Economics Department, The Ronald O Perelman Center for Political Science and Economics, University of Pennsylvania, Philadelphia, PA USA; 3 UNICEF, New York, NY USA; 4 Data and Analytics Section, Division of Data, Analytics, Planning and Monitoring UNICEF, New York, NY USA; 5 Faculty of Education, The University of Hong Kong, Pok Fu Lam, Hong Kong; 6 Harvard Graduate School of Education, Harvard University, Cambridge, MA USA; 7 Department of Child and Adolescent Health and Development, World Health Organization, Geneva, Switzerland; 8 World Bank Group, Washington, DC USA; 9 Department of Epidemiology & Public Health, Swiss Tropical and Public Health Institute, University of Basel, Basel, Switzerland; 10 Faculty of Law, University of Oxford, Oxford, UK; 11 WORLD Policy Analysis Center, Fielding School of Public Health, University of California, Los Angeles, California USA; 12 Inter-American Development Bank, Washington, DC USA; 13 Division of Global Health Equity, Brigham and Women’s Hospital/Harvard Medical School, Boston, MA USA; 14 Early Childhood Development Action Network, Washington, DC USA; 15 University of Nebraska Medical Center, Omaha, NE USA; 16 Department of Child and Adolescent Psychiatry, University of Oxford, Oxford, UK; 17 University of Buenos Aires, Bulnes, Argentina; 18 Global TIES for Children, New York University, New York, NY USA

**Keywords:** Economics, Education

## Abstract

A recent *Nature* article modelled within-country inequalities in primary, secondary, and tertiary education and forecast progress towards Sustainable Development Goal (SDG) targets related to education (SDG 4). However, their paper entirely overlooks inequalities in achieving Target 4.2, which aims to achieve universal access to quality early childhood development, care and preschool education by 2030. This is an important omission because of the substantial brain, cognitive and socioemotional developments that occur in early life and because of increasing evidence of early-life learning’s large impacts on subsequent education and lifetime wellbeing. We provide an overview of this evidence and use new analyses to illustrate medium- and long-term implications of early learning, first by presenting associations between pre-primary programme participation and adolescent mathematics and science test scores in 73 countries and secondly, by estimating the costs of inaction (not making pre-primary programmes universal) in terms of forgone lifetime earnings in 134 countries. We find considerable losses, comparable to or greater than current governmental expenditures on all education (as percentages of GDP), particularly in low- and lower-middle-income countries. In addition to improving primary, secondary and tertiary schooling, we conclude that to attain SDG 4 and reduce inequalities in a post-COVID era, it is essential to prioritize quality early childhood care and education, including adopting policies that support families to promote early learning and their children’s education.

## Introduction

In an important recent *Nature* article, Friedman et al.^1^ modelled within-country inequalities in primary, secondary, and tertiary education and forecast progress towards education-related targets of the Sustainable Development Goals (SDGs). They found that most countries are on track to achieve near-universal primary education by 2030 and schooling gender gaps are closing, but parts of sub-Saharan Africa, North Africa and the Middle East still lag far behind. Progress in secondary education is less promising, with only 10% of adolescents in poorer countries completing 12 schooling grades. An Editorial on the paper (*Education must fix its data deficit)*^2^ notes that data on disparities have played substantial roles in driving gains achieved to date^1^. It calls for more data to identify which groups of children need most help, and urges further progress in tracking what children learn in addition to their completed schooling grades.

SDG 4’s goal is to ensure inclusive and equitable quality education and promote lifelong learning opportunities for all. Friedman et al.’s^1^ paper addresses two SDG 4 targets: 4.1 (free, equitable and quality primary and secondary education) and part of 4.3 (ensuring that men and women have equal access to affordable and quality tertiary education). However, their paper entirely overlooks Target 4.2, which states that by 2030, all girls and boys should have access to quality early childhood development, care and pre-primary education so that they are ready for primary education. As we enter the last decade of the SDG agenda, it is crucial that we hold the world accountable for achieving this target because it is foundational to all learning and the achievement of SDG 4 in totality. SDG 4.2 can only be achieved by collecting and analysing data to track progress and disparities in early-life education, and highlighting governmental actions to accelerate progress by addressing gaps.

The 1990 Jomtien World Declaration on Education for All^3^ stated that “Learning begins at birth”, and the importance of child development in preschool years has been included in all international declarations since, including the 2000 Dakar Framework for Action: Education for All^4^, the Millennium Development Goals and the SDGs. Given strong evidence that foundations for adolescent and adult human capital are established in the early years, we can no longer consider education to begin when children start primary school. It is critical to bear in mind the long-term importance of the enormous learning that occurs—or does not occur—from before birth to when children walk into their first-grade classrooms. Inequalities are evident from the start and generally very large by the time children enter formal schooling systems.

Promoting early learning outcomes and mitigating inequities requires tracking children’s progress or development from their very first years of life. SDG 4.2 indicators focus on the proportion of children aged 24–59 months who are developmentally on track in health, learning and psychosocial wellbeing, by sex (4.2.1) and participation rates in organized learning (1 year before the official primary entry age), by sex (4.2.2)^5^. The 2020 UN Secretary General’s Report^6^, based on 74 countries with comparable 2011–2019 data, states that ~70% of children 3–4 years of age are on track developmentally in at least three of the following domains: literacy-numeracy, physical development, social-emotional development and learning. Participation in organized learning programmes 1 year before the official age of primary school entry grew steadily from 62% in 2010 to 67% in 2018. Variation among countries remains wide^7^, with values ranging from 9% to nearly 100%. Of 16 countries with trend data since 2010, the largest progress was observed in Iraq, Laos and Sierra Leone, but no progress or even reduced coverage in Cameroon, Chad or Swaziland^7^. Further, large socioeconomic and rural-urban within-country disparities are found for preschool children^8^.

### The importance of early childhood development at home, child day care and in pre-primary education

The evidence is incontrovertible: learning begins at and even before birth. Brain development is extremely rapid and learning takes place as children interact with adults who facilitate, name and interpret their experiences. Children’s brain volumes double during their first year and reach 80–90% of their adult sizes by age 3^9^, and learning progresses rapidly across all modalities^10,11^. For example, foetuses and newborns distinguish their mothers’ voices from others^12^ and, within days after birth, associate auditory and visual information together, such as mothers’ voices with their faces^13^.

Not only are children actively learning about people and objects around them from birth, but they are learning *how* to learn, mainly from other people. Child-directed speech, emotional attunement between caregivers and children that promotes affection and trust, and predictable adult responsiveness to children’s communication are foundations of children’s learning^14^. The importance of these elements for young children’s development is articulated in the Nurturing Care Framework (NCF), developed in follow-up of the 2017 *Lancet* series *Advancing Early Childhood Development: From Science to Scale*^15^. The NCF describes the qualities of holistic environments that promote, support and protect young children’s health, nutrition, safety, and early learning, and satisfy the need for warm and affectionate responsiveness from others.

Clearly, the elements for success in school and lifelong learning are developed long before children enter primary schools. Both stimulating home environments and participation in high-quality early childcare and educational programmes independently and interactively support children’s early learning. One or more years of quality pre-primary education builds cognitive and social skills founded on the substantial learning that takes place through interactions with familiar adults and other children at home, as well as in child day care.

Poverty and undernutrition mar early development for far too many children, estimated at 250 million, or 43%, of all children under 5 years old in low- and middle-income countries (LMICs)^16,17^. These early disadvantages put children at risk of inadequate learning, incomplete schooling and lower adult earnings^18–20^. The average percentage losses of adult income resulting from loss of schooling due to stunting or living in extreme poverty in early life are estimated to be about 27%^21^. Early disadvantages are compounded by poor quality and high out-of-pocket costs of early childcare and educational and pre-primary programmes. Both poverty and stunting can be mitigated by governmental actions. For example, both minimum wage and parental leave policies have been shown to improve nutrition, family income, and healthy child development^22,23^.

### Unequal opportunities from the start

Inequalities in learning and development are evident early. Analysis of data collected since 2010 through 135 nationally representative datasets (primarily Demographic Health Surveys & Multiple Indicator Cluster Surveys) showed that risks for early childhood development and opportunities for early learning varied widely across regions^7^. All four indicators analysed showed clear gradations of increasing disadvantage for young children from upper-middle- to lower-middle- to low-income countries. From 16% to 36% to 55% of children under age five were exposed to extreme poverty or stunting; 15% to 38% to 46% of 3- to 4-year-olds were not receiving basic stimulation for learning at home; 13% to 26% to 40% of 3- to 4-year-olds were not developmentally on track (as measured by the Early Childhood Development Index, or ECDI), and from 47% to 63% to 79% of children of the same age were not attending early childcare and educational programmes. Gradations of disadvantage were found also within countries with respect to household wealth and rural versus urban residence, with poor rural households having the greatest disadvantages. The differences between boys and girls on the four indicators were either small or non-significant, with slight advantages for girls on stunting and ECDI^7^. Consistent with the schooling data reported by Friedman et al.^1^, children in sub-Saharan Africa were most likely at risk due to poverty and stunting, had the lowest percentages receiving adequate stimulation at home (47% vs. 69% for the average of 62 countries from different regions), the smallest proportion developmentally on track in terms of the ECDI (61% vs. 75% for the overall average), and the lowest percentages attending some form of early care and educational programmes (24% vs. 39% for the overall average).

At least 95% of children between 4 years of age and entry into compulsory primary school participate in pre-primary programmes in the 28 European Union countries, reaching the target set in their Strategic Framework for Cooperation in Education and Training^24^. Pre-primary programme enrolments globally have increased dramatically from 35% in 2000 to over 62% in 2019, with increases in all regions. In LMICs, enrolments nearly doubled over this period. For example, enrolments increased from 9 to 20% in low-income countries and from 45 to 76% in upper-middle-income countries. However, substantial gaps remain, between and within countries and between urban and rural areas and by socioeconomic status. For example, the 2019 pre-primary programme gross enrolment rate was only 32% in sub-Saharan Africa^25^ compared to 62% globally.

Governments vary in their provision of pre-primary programmes. Among 194 countries, 68 countries have legal mandates for either free and/or compulsory pre-primary education. Among these 68 countries, pre-primary education is free and compulsory in 46 countries. Notably, legal provisions for free pre-primary education exist in 3/27 low-income countries, 11/34 lower-middle-income countries, 23/33 upper-middle-income countries, and 24/26 high-income countries. There is thus a gradient between income and legal provision for pre-primary education. On the one hand, countries that legislated either free and/or compulsory education saw their enrolments increase from 41.4% in 1999 to 82.8% in 2018. On the other hand, countries with no legal frameworks for pre-primary education increased from 52.9% in 1999 to 63% in 2018^26^. Countries offering 1 year of tuition-free pre-primary programming, had 16% higher gross enrolment rates compared to countries without tuition-free pre-primary provision^27^. Countries providing at least 1 year of free and compulsory pre-primary programming had 10% higher primary school completion rates^27^, suggesting that free compulsory programmes can set children on paths to longer-term educational attainment.

Inequalities in both provision of and access to early learning opportunities accumulate and extend as children progress through pre-primary, primary and secondary schooling^28^. This lessens children’s chances of catching-up and of realizing the global community’s efforts to eliminate documented inequalities in schooling^1^.

### Early disadvantage is costly

Learning occurs progressively and skills build on each other. Complementarities between skills increase motivation and make learning at later ages easier. A recent national longitudinal study showed dynamic complementarity between access to pre-primary education and improved primary and secondary education, with access to both particularly beneficial in terms of increased educational attainment and earnings for children from more disadvantaged households^29^. Moreover, recent evidence indicates that universally provided high-quality early care and education programmes reduce learning gaps between children from higher and lower socioeconomic status households^30^.

Given this, it is unsurprising that longitudinal studies show strong evidence for cognitive, social, and economic returns to high-quality early care and educational programmes^30^. For example, expanded pre-primary programme attendance for Argentinian children aged 3–5 increased primary school language and mathematics scores by 0.3 and 0.2 standard deviations (SD), respectively, for both boys and girls^31^. Adults in 12 LMICs who had attended early care and education programmes stayed in school on average 0.9 years longer, controlling for family background and other factors^32^.

We undertook new analyses of the value of pre-primary school, using a sample of 430,000 children from 73 middle- and high-income countries (Supplementary Table 1) surveyed in the 2018 *Programme for International Student Assessments* (PISA)^33^.

## Results

**Fig. 1:**
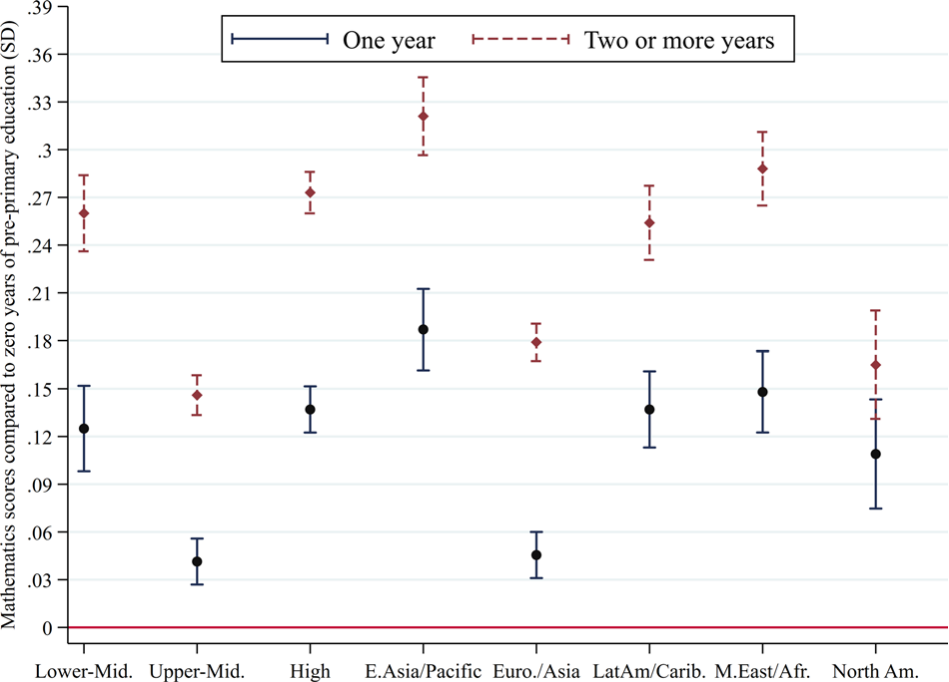
Estimated average differences in mathematics test scores at age 15 between pre-primary programme participants and non-participants. Notes: Mathematics scores in standard deviations (SD) and confidence intervals for students with 1 year of pre-primary attendance compared to 2 or more years of pre-primary attendance by country income and regional groupings. All empirical models are based on 2018 PISA data and control for child sex and age, age of school entry, fathers’ and mothers’ schooling attainment and household socioeconomic status. Supplementary Table 2 presents additional details.

**Fig. 2:**
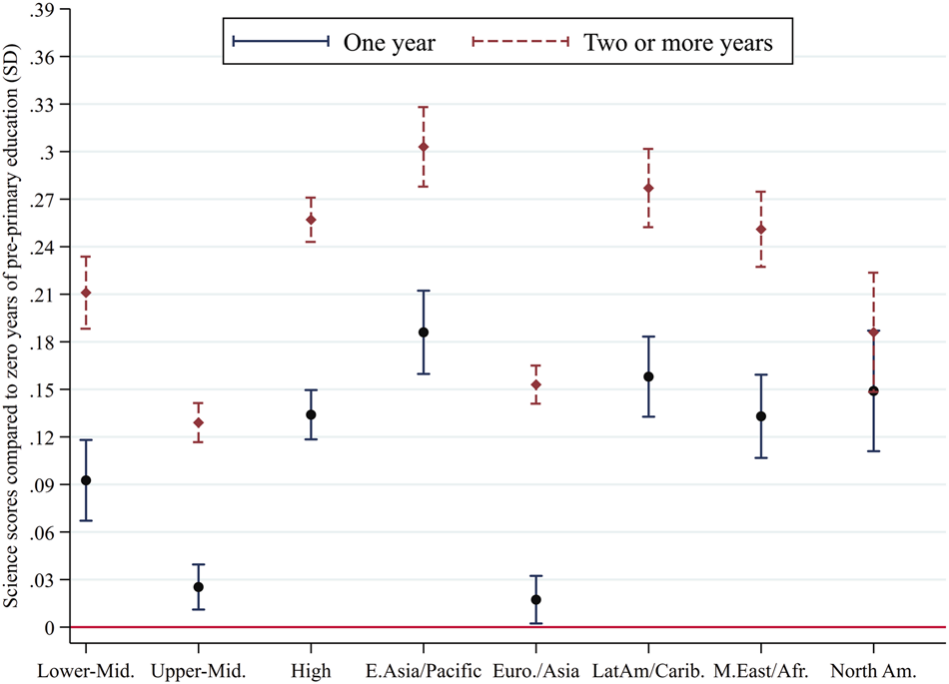
Estimated average differences in science test scores at age 15 between pre-primary programme participants and non-participants. Notes: Science scores in standard deviations (SD) and confidence intervals for students with 1 year of pre-primary attendance compared to 2 or more years of pre-primary attendance by country income and regional groupings. All empirical models are based on 2018 PISA data and control for child sex and age, age of school entry, fathers’ and mothers’ schooling attainment and household socioeconomic status. Supplementary Table 3 presents additional details.

Students who participated in 1 year of pre-primary programmes had on average 0.10 SD (95% CI 0.09, 0.11) higher mathematics test scores at age 15 compared to students who participated in <1 year or no pre-primary programmes; students who participated in 2 or more years had on average 0.22 SD (95% CI 0.21, 0.23) higher scores than their non-participating peers, controlling for a range of basic sociodemographic characteristics. Estimated associations were almost identical for science scores, with increments of 0.09 SD (95% CI 0.08, 0.10) for at least 1 year, and 0.20 SD (95% CI 0.19, 0.21) for 2 or more years of pre-primary programme exposure. Figures 1 and 2 show that these associations were slightly larger for lower-middle- (*N* = 6) and high-income (*N* = 43) countries than for upper-middle-income countries (*N* = 24). The largest associations were found in the East Asia and Pacific region (*N* = 11), while associations were weakest in Europe and Central Asia and in North America. Full results are presented in Supplementary Table 2 (mathematics) and Supplementary Table 3 (science). Although these analyses control for potential confounding variables such as household socioeconomic status, it is important to recognize that they are non-causal in nature. Yet, our results are largely robust, including more conservative models with fixed effects that aim to capture either major geographical regions or school types (Supplementary Table 4), and are consistent with causal estimates of the benefits of pre-primary education on cognitive outcomes (*d* = 0.20–0.35)^34^.

### Costs of inaction

One corollary of lifelong benefits of investments in the early years is that inadequate investments incur significant future costs. To illustrate one important component of the costs of not achieving SDG 4.2.2, we simulated the costs of inaction (COI), or the present discounted value of the losses in future income (net of pre-primary school costs) if pre-primary programme enrolments remain at their 2018 levels instead of becoming universal^21^ (see Supplementary Table 5). We note that preschool education is associated with other short- and long-term impacts that are not included in the model, such as female labour participation or reduced crime in a society. The omission of such benefits in our estimates means that they possibly are conservative, and underestimate the true benefits of early educational programmes. On the other hand, general equilibrium effects may mean that the rates of return to preschool would decline with expansion, which would tend to work in the opposite direction.

**Fig. 3:**
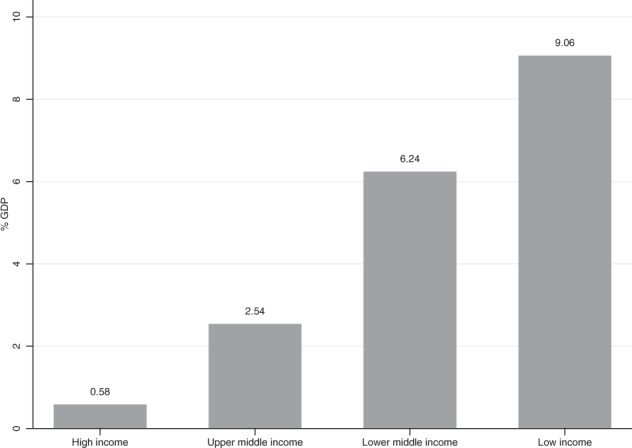
Median COIs for not reaching SDG 4.2.2 for 1 year. Median simulated costs of inaction in terms of percentage of GDP loss of not reaching universal coverage for pre-primary programmes by World Bank country income group classification.

**Fig. 4:**
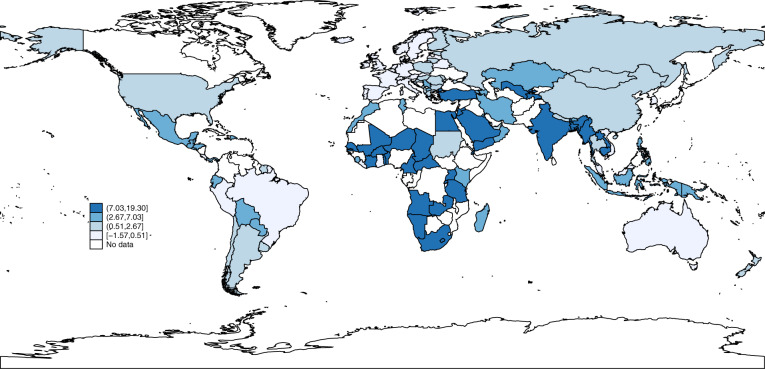
Cost of inaction in terms of percentage of GDP loss of not reaching universal coverage for pre-primary programmes by country. Note: Children are assumed to enter the labour market at age 18 and 8% benefits are captured for 45 years, calculated with discount rate = 3%.

The 134 countries with available data together have populations of over 6.3 billion people. Supplementary Table 5 presents the 2018 UNESCO gross pre-primary enrolment rates, the COI due to shortfalls from the SDG 4.2.2 for individual countries, and sensitivity analysis to explore the uncertainty regarding the value of the main parameters in the simulation. Figure 3 gives the median COIs for not reaching SDG 4.2.2 for 1 year for country groups: high (0.58% of GDP), upper-middle (2.54% of GDP), lower-middle (6.24% of GDP) and low-income (9.06% of GDP). The association with income is inverse, with COI tending to be greater in lower-income countries. These are considerable, particularly in lower-income countries where estimated losses often exceed the annual governmental expenditures on all levels of education, which are 4.1% for low-income countries, 4.4% for lower-middle-income counties, 4.3% for upper-middle-income countries and 5% for high-income countries. Figure 4 shows the COI by country. Most countries with the highest COI relative to GDP are in sub-Saharan Africa and Asia.

## Discussion

According to UNESCO’s educational expenditure data in 84 countries, most LMIC governments spent under 5% of their educational budgets on pre-primary programmes. Because of limited resources, external aid plays an important role, but donor contributions to pre-primary programmes comprised only about 2% of their spending on basic education in 2014^35^, suggesting little attention to pre-primary programmes from international donors. Because of these low investments, many LMIC households bear substantial financial burdens. For example, household out-of-pocket payments accounted for 63% of total spending on pre-primary programmes in Nepal^36^ and 100% in Uganda^37^. Current limited governmental and donor investment in pre-primary programmes therefore contributes to existing inequalities.

We can no longer consider education to begin when children start their first grade at school. Learning begins before birth, and the quality of individuals’ early learning and development has marked effects on their later educational achievement and human capital more generally. There are known policies, such as paid parental leave^38^, minimum wages^23^, tuition-free pre-primary programmes^23,27,38^, and income support for the poorest families^39^ that—together with improved parental support and provision of early care and educational programmes—can fundamentally change children’s trajectories through school. Inequalities in grade completion and, importantly, inequalities in what children learn in the grades they do complete, will be addressed more effectively if interventions and investments prioritise children’s pre-primary learning as a continuum with primary and secondary schooling^40,41^.

We have shown that the benefits of pre-primary programme learning are high, both for individuals and for the economies of their countries, and the COI are high as well. During the 9 years until 2030, it is crucial that investments in early childhood development and learning are given high priority. Investments are particularly necessary during and post-COVID-19, when millions of children are at risk of deprivation during their crucial months and years of early development. COVID-19 will slow down progress on the SDGs and in particular SDG 4.2 as pre-primary programmes are closed, poverty levels rise, and inequalities are amplified^42^. One recent study^43^ simulated losses due to pre-primary programme closures because of the COVID-19 pandemic on future earnings when current preschool-age children become adults for 140 countries. Closures of preschool for 6 months led to estimated losses in future earnings equivalent to around 2.5% of GDP. Another study, using data from 196 countries estimate that global 12-month closures to early childcare and education services will result in more than 22 million additional children falling behind in their development, with adverse consequences for learning in adolescence and earnings in adulthood^44^. Families are struggling with job losses and financial crises, and the mental and social wellbeing of parents and their young children are at risk^42^. Funding, policies, programme quality, and data will all suffer, increasing the COI. In sum, we believe that there is an undeniable case for efforts to improve early childhood development and learning to be made front and centre as countries struggle through competing priorities to recovery.

## Methods

### Pre-primary attendance and adolescent mathematics and science test scores

We used data for 430,264 adolescents in 73 middle- and high-income countries surveyed in the 2018 *Programme for International Student Assessments* (PISA). The PISA is an international programme to assess adolescents’ reading, mathematics, and science literacy every 3 years for nationally representative samples of 15-year-old students enroled in school. The PISA also collects data on the characteristics of adolescents and their backgrounds. Among such information, the PISA asks students to retrospectively report how many years of pre-primary education they attended, following the International Standard Classification of Education Level 0 (ISCED-0).

Using PISA data, we used multivariate regression models to assess the association between years of pre-primary education attendance and mathematics and science test scores. Given that PISA employs an imputation methodology to provide plausible values for each student’s test score, as a first step, we averaged such plausible values to obtain a single mathematics and science test score, which we standardized to have a mean of zero and standard deviation of one to aid interpretability. Subsequently, we estimated two models to test the association between students’ mathematics and science standardized test scores (*zTestScore*_*i*_) and binary variables indicating whether students attended one year (*OneYear*_*i*_) or two or more years (*TwoYear*_*i*_) of pre-primary education.

$$zTestScore_i = \alpha + \beta _1 \ast OneYear_i + \beta _2 \ast TwoYear_i + + Covar_i \ast \theta + \mu _i$$

We added covariates to the model in order to reduce potential bias, including adolescents’ age and gender, a wealth index provided by PISA, maternal and paternal education, and age of entry to primary school. Furthermore, we included country and subnational (geographical region or school type) fixed effects to make within-country comparisons.

We estimated separate models according to the World Bank’s categorization of income groups, i.e., for lower-middle-income countries (*N* = 6), upper-middle-income countries (*N* = 24), and high-income countries (*N* = 43), and region, i.e., East Asia and Pacific (*N* = 11), Europe and Central Asia (*N* = 42), Latin America and the Caribbean (*N* = 10), Middle East and North Africa (*N* = 8), and North America (*N* = 2).

### Cost of inaction

To obtain the COI for not reaching the SDG 4.2 targets for 1 year, we extend a procedure used to estimate the COI related to pre-primary schooling for five Latin American countries for the *Lancet* series on early childhood development^21,43,45,46^. In Eq. (1), the increase in individual earnings in future decades as a consequence of participating in preschool (*PCI*_*j*_ x *i*, where *PCI*_*j*_ is per capita income in year *j* and *i* is the causal impact of preschool on that income) is discounted by the discount rate *d* and summed over the relevant years in which earnings are expected to be affected (from when the individual starts to work *a* years after preschool through *t* years of working life) and then compared with the per child programme cost (*c*) for the *N* children covered by preschool (i) for the 2018 enrolments and (ii) if the SDG 4.2 targets were attained.

1 $${\mathrm{COI}} = \left( {\mathop {\sum}\nolimits_{j = a}^{t + a} {\frac{{PCI_j \times i}}{{\left( {1 + d} \right)^j}}} - c} \right) \times N$$

The COI for each country therefore depends on projections for that country’s *PCI*, the impact of preschool on per capita income (*i*), the per child programme cost (*c*) and the expansion in enrolment in order to obtain the SDG 4.2 targets (100%−*N*).

Note that this procedure probably leads to conservative estimates of the COI because only effects on adult earnings are included, but other short- and long-term impacts that are hard to monetize, such as reduced crime, are omitted. On the other hand, there may be general equilibrium effects that work in the opposite direction. Information on the discount rate (*d*), number of children affected (*N*), impact of pre-primary school on adult earnings (*i*) and cost (*c*) of pre-primary is critical for the simulations. As is common for many evaluations of social programmes where benefits accrue in the long term, in our simulations we used a discount rate (*d*) of 3%. For the current enrolments (*N*) we used the 2018 gross enrolment rates (GER) reported in UNESCO, Institute for Statistics^47^. For the SDG-targeted enrolments we used the maximum of 100% and the actual 2018 enrolments. The latter may be over 100% because the UNESCO data estimate the total enrolments of pre-primary students of all ages to the ratio of children in the country of pre-primary school ages, and there is catch-up in some countries with older children attending pre-primary school. We assume that such catch-up is of interest in attaining the SDG 4.2 targets. This use of the UNESCO GERs causes an underestimate of the COIs. The causal evidence on long-term effects of pre-primary school from randomized experiments in which children have been subsequently followed-up in their adult years is sparse, but suggests that impacts in earnings are substantial, of the order of 14% over the lifetime^48^. However, since that evidence comes from high-quality small-scale interventions targeting children from low socioeconomic backgrounds, it may not be externally valid in the case of lower-quality programmes, programmes implemented at scale or with children participating from all socioeconomic backgrounds. Thus, we have adopted a lower impact value of 8% for our simulations. The estimates for per child costs of existing pre-primary services, *c*, vary considerably across countries^49,50^. Since an important part of these variations reflects differences in wages and prices for services that relate to income levels across countries, we adjusted the programme costs for each group of countries based on the price level ratio of purchasing-power-parity (PPP) conversion factors that reflect the value of wages for services better than do market exchange rates. These assumptions are strong so the COI estimates are somewhat crude for any particular country. Supplementary Table 5 provides sensitivity analyses. COI do not change substantially if the assumed impact of pre-primary school on earnings decreases one or two percentage points, or if costs increase by 10% or 20%. If we apply higher discount rates (from 4% to 5%), patterns are similar but with smaller COI. Even though estimates would probably need to be refined to provide guidance for any particular country’s polices, they provide a useful order of magnitude for understanding an important component of the long-run global economic costs of lags in reaching the SDG 4.2 targets.

### Reporting summary

Further information on research design is available in the Nature Research Reporting Summary linked to this article.

## Supplementary information

Supplementary Information

Reporting Summary

## Data Availability

This study used data that are available from public online repositories, most of which require a straightforward registration process and usage agreement with the data provider. The 2018 PISA data are available here: https://www.oecd.org/pisa/data/2018database/#d.en.516012 and the gross enrolment ratio and educational expenditure data are available here: http://data.uis.unesco.org/. The authors may be contacted for analytic datasets and for assistance in acquiring data for the replication of this study. All maps presented in this study have been produced by the authors and no permissions are required for publication.

## Code availability

All code used for these analyses is available from the authors.
