# Supplementary material for: Measuring and forecasting progress in education: what about early childhood?
Source: NPJ Sci Learn. 2021 Sep 10;6:27. doi: 10.1038/s41539-021-00106-7 (PMC8433172; doi:10.1038/s41539-021-00106-7)
Supplement: Supplementary file 1 — Supplementary Information [file 41539_2021_106_MOESM1_ESM.pdf]

Supplementary Information

Supplementary Table 1: PISA countries

East Asia and Pacific

Country	*N*
Australia	9,901
Brunei Darussalam	4,563
Indonesia	9,202
Japan	4,849
Korea	5,306
Malaysia	5,610
New Zealand	4,970
Philippines	5,985
Singapore	4,949
Thailand	7,601
Vietnam	4,725

Latin America and the Caribbean

Country	*N*
Argentina	10,351
Brazil	7,609
Chile	5,512
Colombia	5,775
Costa Rica	5,215
Dominican Republic	4,175
Mexico	5,892
Panama	5,017
Peru	5,382
Uruguay	4,523

Middle East and North Africa

Country	*N*
Israel	5,320
Jordan	6,690
Lebanon	3,008
Malta	2,767
Morocco	5,212
Qatar	8,662
Saudi Arabia	2,257
United Arab Emirates	13,279

North America

Country	*N*
Canada	17,004
United States	2,892

Europe and Central Asia

Country	*N*
Albania	5,487
Austria	5,398
Belarus	4,839
Belgium	6,641
Bosnia and Herzegovina	2,390
Bulgaria	4,230
Croatia	4,606
Czech Republic	5,774
Denmark	5,866
Estonia	4,091
Finland	4,536
France	4,875
Georgia	4,016
Germany	3,745
Greece	5,354
Hungary	4,341
Iceland	2,401
Ireland	4,394
Italy	9,591
Kazakhstan	10,066
Kosovo	3,083
Latvia	3,935
Lithuania	4,508
Luxembourg	3,870
Moldova	4,421
Montenegro	4,130
Netherlands	3,891
Norway	4,096
Poland	3,870
Portugal	4,745
Romania	4,079
Russian Federation	5,609
Serbia	5,536
Slovak Republic	4,997
Slovenia	4,756
Spain	30,664
Sweden	4,204
Switzerland	4,797
Turkey	4,000
Ukraine	4,234
United Kingdom	10,408

Supplementary Table 2: Estimated associations between pre-primary programme attendance and mathematics test scores at age 15

Variables	Lower middle income	Upper middle income	High income	East Asia and Pacific	Europe and Central Asia	Latin America and the Caribbean	Middle East and North Africa	North America
**Pre-primary attendance**								
One	0.125***	0.041***	0.137***	0.187***	0.046***	0.137***	0.148***	0.109***
	(0.013)	(0.007)	(0.007)	(0.013)	(0.007)	(0.012)	(0.013)	(0.018)
Two or more	0.260***	0.146***	0.273***	0.321***	0.179***	0.254***	0.288***	0.165***
	(0.012)	(0.006)	(0.007)	(0.012)	(0.006)	(0.012)	(0.01)	(0.017)
Female	0.042***	-0.032***	-0.051***	0.017***	-0.053***	-0.114***	0.071***	-0.056***
	(0.008)	(0.004)	(0.003)	(0.007)	(0.003)	(0.006)	(0.007)	(0.011)
Age	0.088***	0.114***	0.201***	0.163***	0.165***	0.165***	0.146***	0.183***
	(0.015)	(0.007)	(0.006)	(0.012)	(0.006)	(0.010)	(0.013)	(0.020)
**Wealth quintile (omitted category is Q1)**								
Q2	0.336***	0.218***	0.267***	0.288***	0.204***	0.223***	0.211***	0.274***
	(0.012)	(0.006)	(0.008)	(0.011)	(0.006)	(0.007)	(0.013)	(0.041)
Q3	0.467***	0.328***	0.379***	0.487***	0.290***	0.356***	0.331***	0.438***
	(0.015)	(0.007)	(0.008)	(0.013)	(0.007)	(0.009)	(0.014)	(0.039)
Q4	0.572***	0.414***	0.449***	0.620***	0.352***	0.489***	0.426***	0.491***
	(0.019)	(0.008)	(0.008)	(0.013)	(0.007)	(0.011)	(0.014)	(0.038)
Q5	0.606***	0.465***	0.422***	0.660***	0.347***	0.668***	0.270***	0.475***
	(0.029)	(0.010)	(0.008)	(0.014)	(0.008)	(0.014)	(0.014)	(0.037)
**Father's education (omitted category is pre-primary)**								
Primary	-0.027	0.058***	0.171***	0.028	0.231***	0.068***	0.003	0.018
	(0.022)	(0.014)	(0.016)	(0.023)	(0.019)	(0.014)	(0.025)	(0.124)
Lower secondary	-0.001	0.085***	0.210***	0.073***	0.268***	0.097***	-0.004	0.000
	(0.021)	(0.013)	(0.014)	(0.023)	(0.017)	(0.013)	(0.022)	(0.117)
Upper secondary	0.151***	0.229***	0.320***	0.154***	0.415***	0.187***	0.145***	-0.072
	(0.027)	(0.015)	(0.015)	(0.027)	(0.017)	(0.018)	(0.025)	(0.116)
Post-secondary, non-tertiary	0.151***	0.181***	0.340***	0.172***	0.418***	0.185***	0.090***	0.147
	(0.020)	(0.013)	(0.014)	(0.022)	(0.017)	(0.013)	(0.021)	(0.113)
Short-cycle tertiary	0.091***	0.176***	0.340***	0.222***	0.386***	0.155***	0.140***	0.198*
	(0.024)	(0.014)	(0.014)	(0.025)	(0.017)	(0.015)	(0.023)	(0.114)
Bachelor's or equivalent	0.161***	0.320***	0.534***	0.378***	0.553***	0.270***	0.369***	0.395***
	(0.022)	(0.013)	(0.014)	(0.023)	(0.017)	(0.014)	(0.021)	(0.113)

Variables	Lower middle income	Upper middle income	High income	East Asia and Pacific	Europe and Central Asia	Latin America and the Caribbean	Middle East and North Africa	North America
**Mother's education (omitted category is pre-primary)**								
Primary	-0.067***	0.035**	0.092***	-0.028	0.161***	0.049***	-0.006	-0.352**
	(0.022)	(0.014)	(0.016)	(0.025)	(0.019)	(0.015)	(0.023)	(0.139)
Lower secondary	-0.106***	0.033**	0.087***	-0.026	0.158***	0.067***	-0.113***	-0.365***
	(0.021)	(0.014)	(0.015)	(0.024)	(0.017)	(0.015)	(0.020)	(0.131)
Upper secondary	0.044	0.106***	0.190***	0.063**	0.285***	0.111***	-0.027	-0.362***
	(0.029)	(0.016)	(0.015)	(0.030)	(0.018)	(0.019)	(0.025)	(0.130)
Post-secondary, non-tertiary	-0.024	0.142***	0.284***	0.028	0.361***	0.214***	0.016	-0.133
	(0.020)	(0.014)	(0.014)	(0.023)	(0.017)	(0.014)	(0.019)	(0.127)
Short-cycle tertiary	-0.022	0.162***	0.306***	0.110***	0.371***	0.201***	0.061***	-0.088
	(0.024)	(0.014)	(0.015)	(0.026)	(0.017)	(0.016)	(0.022)	(0.127)
Bachelor's or equivalent	-0.018	0.255***	0.432***	0.110***	0.521***	0.247***	0.133***	0.016
	(0.022)	(0.014)	(0.014)	(0.024)	(0.017)	(0.015)	(0.020)	(0.127)
Age of school entry	-0.172***	-0.141***	-0.152***	-0.146***	-0.158***	-0.149***	-0.173***	-0.060***
	(0.006)	(0.003)	(0.002)	(0.004)	(0.003)	(0.003)	(0.004)	(0.006)
Observations	31,661	140,459	258,144	60,007	241,195	57,928	50,680	20,454
R-squared	0.274	0.237	0.264	0.443	0.223	0.300	0.224	0.114

Notes: Standard errors in parentheses. Based on the 2018 PISA data. Estimates represent standardized increases in normalized test scores.

*** p<0.01, ** p<0.05, * p<0.1

Supplementary Table 3: Estimated associations between pre-primary programme attendance and science test scores at age 15

Variables	Lower middle income	Upper middle income	High income	East Asia and Pacific	Europe and Central Asia	Latin America and the Caribbean	Middle East and North Africa	North America
**Pre-primary attendance**								
One	0.093***	0.025***	0.134***	0.186***	0.017**	0.158***	0.133***	0.149***
	(0.013)	(0.007)	(0.008)	(0.013)	(0.008)	(0.013)	(0.013)	(0.019)
Two or more	0.211***	0.129***	0.257***	0.303***	0.153***	0.277***	0.251***	0.186***
	(0.01)	(0.006)	(0.007)	(0.013)	(0.006)	(0.013)	(0.012)	(0.019)
Female	0.066***	0.055***	0.037***	0.028***	0.034***	-0.044***	0.231***	0.023*
	(0.008)	(0.004)	(0.003)	(0.007)	(0.003)	(0.006)	(0.008)	(0.013)
Age	0.072***	0.114***	0.186***	0.127***	0.153***	0.184***	0.134***	0.188***
	(0.014)	(0.007)	(0.006)	(0.012)	(0.006)	(0.011)	(0.013)	(0.022)
**Wealth quintile (omitted category is Q1)**								
Q2	0.265***	0.215***	0.266***	0.245***	0.192***	0.214***	0.197***	0.294***
	(0.011)	(0.005)	(0.009)	(0.011)	(0.007)	(0.008)	(0.014)	(0.045)
Q3	0.383***	0.306***	0.361***	0.422***	0.259***	0.336***	0.315***	0.455***
	(0.014)	(0.006)	(0.009)	(0.013)	(0.007)	(0.010)	(0.014)	(0.043)
Q4	0.471***	0.374***	0.414***	0.541***	0.303***	0.442***	0.400***	0.493***
	(0.018)	(0.008)	(0.009)	(0.014)	(0.007)	(0.012)	(0.015)	(0.042)
Q5	0.475***	0.395***	0.359***	0.552***	0.271***	0.580***	0.214***	0.457***
	(0.028)	(0.010)	(0.009)	(0.014)	(0.008)	(0.015)	(0.014)	(0.041)
**Father's education (omitted category is pre-primary)**								
Primary	0.014	0.070***	0.148***	0.019	0.234***	0.063***	0.020	-0.181
	(0.021)	(0.014)	(0.017)	(0.024)	(0.019)	(0.014)	(0.025)	(0.137)
Lower secondary	0.001	0.080***	0.205***	0.027	0.272***	0.082***	0.025	0.010
	(0.020)	(0.013)	(0.015)	(0.023)	(0.018)	(0.014)	(0.023)	(0.129)
Upper secondary	0.188***	0.235***	0.329***	0.191***	0.426***	0.173***	0.143***	0.029
	(0.025)	(0.015)	(0.016)	(0.028)	(0.018)	(0.019)	(0.026)	(0.129)
Post-secondary, non-tertiary	0.170***	0.198***	0.346***	0.166***	0.431***	0.194***	0.093***	0.189
	(0.019)	(0.013)	(0.015)	(0.022)	(0.017)	(0.014)	(0.021)	(0.125)
Short-cycle tertiary	0.115***	0.208***	0.351***	0.233***	0.412***	0.153***	0.184***	0.187
	(0.022)	(0.014)	(0.015)	(0.025)	(0.018)	(0.016)	(0.023)	(0.126)
Bachelor's or equivalent	0.154***	0.330***	0.526***	0.356***	0.556***	0.271***	0.397***	0.370***
	(0.021)	(0.013)	(0.015)	(0.023)	(0.017)	(0.015)	(0.022)	(0.126)

Variables	Lower middle income	Upper middle income	High income	East Asia and Pacific	Europe and Central Asia	Latin America and the Caribbean	Middle East and North Africa	North America
**Mother's education (omitted category is pre-primary)**								
Primary	-0.105***	0.059***	0.117***	-0.022	0.216***	0.059***	-0.027	-0.484***
	(0.021)	(0.014)	(0.018)	(0.025)	(0.020)	(0.016)	(0.024)	(0.154)
Lower secondary	-0.142***	0.040***	0.110***	-0.033	0.189***	0.072***	-0.121***	-0.417***
	(0.020)	(0.014)	(0.016)	(0.025)	(0.018)	(0.015)	(0.020)	(0.145)
Upper secondary	0.044	0.140***	0.202***	0.085***	0.313***	0.163***	-0.064**	-0.437***
	(0.028)	(0.015)	(0.016)	(0.030)	(0.018)	(0.020)	(0.026)	(0.144)
Post-secondary, non-tertiary	-0.027	0.161***	0.297***	0.044*	0.391***	0.214***	-0.008	-0.229
	(0.019)	(0.013)	(0.015)	(0.024)	(0.018)	(0.015)	(0.020)	(0.140)
Short-cycle tertiary	-0.013	0.181***	0.329***	0.134***	0.411***	0.212***	0.011	-0.179
	(0.023)	(0.014)	(0.016)	(0.026)	(0.018)	(0.017)	(0.022)	(0.141)
Bachelor's or equivalent	-0.022	0.277***	0.449***	0.134***	0.543***	0.309***	0.111***	-0.080
	(0.021)	(0.014)	(0.015)	(0.025)	(0.018)	(0.016)	(0.020)	(0.140)
Age of school entry	-0.149***	-0.127***	-0.149***	-0.146***	-0.146***	-0.141***	-0.161***	-0.072***
	(0.005)	(0.003)	(0.002)	(0.004)	(0.003)	(0.003)	(0.004)	(0.007)
Observations	31,661	140,459	258,144	60,007	241,195	57,928	50,680	20,454
R-squared	0.296	0.209	0.215	0.406	0.225	0.285	0.193	0.072

Notes: Standard errors in parentheses. Based on the 2018 PISA data. Estimates represent standardized increases in normalized test scores.

*** p<0.01, ** p<0.05, * p<0.1

Supplementary Table 4: Estimated associations between pre-primary programme attendance and mathematics and science test scores including stratum fixed effects

	Stratum fixed effects		School fixed effects	
VARIABLES	Mathematics	Science	Mathematics	Science
One	0.0593***	0.0463***	0.0346***	0.0237***
	(0.00483)	(0.00503)	(0.00443)	(0.00467)
Two or more	0.152***	0.129***	0.0893***	0.0682***
	(0.00430)	(0.00448)	(0.00398)	(0.00420)

Notes: Based on the 2018 PISA data. Estimates represent standardized increases in normalized test scores. All empirical models control for child sex and age, age of school entry, fathers' and mothers' schooling attainment, household socioeconomic status, and stratum fixed effects, which capture geographic areas or school type. Numbers displayed are linear estimates with standard errors in parentheses.

Supplementary Table 5: Cost of inaction in terms of % of GDP loss of not reaching universal coverage for pre-primary school by countries grouped by World Bank income groups with sensitivity analysis to changes in the value of key parameters

Group	Country	2018 Gross Enrollment Rates (%)[a]	COI (% GDP)	Sensitivity analysis					
				i=7	*i=6*	*d=4*	*d=5*	*cost +10%*	*cost +20%*
High income	Antigua and Barbuda	0.7	2.0	1.7	1.5	1.5	1.1	2.0	2.0
	Australia	1.6	0.0	0.0	0.0	0.0	0.0	0.0	0.0
	Austria	1.0	0.0	0.0	0.0	0.0	0.0	0.0	0.0
	Bahamas, The	0.3	5.3	4.6	4.0	3.9	2.9	5.3	5.3
	Bahrain	0.5	4.0	3.5	3.0	2.9	2.2	4.0	4.0
	Barbados	0.9	0.8	0.7	0.6	0.6	0.4	0.8	0.8
	Belgium	1.2	0.0	0.0	0.0	0.0	0.0	0.0	0.0
	Brunei Darussalam	0.6	3.5	3.0	2.6	2.5	1.9	3.4	3.4
	Chile	0.8	1.5	1.3	1.1	1.1	0.8	1.5	1.5
	Croatia	0.7	1.9	1.7	1.4	1.4	1.0	1.9	1.9
	Cyprus	0.8	1.6	1.4	1.2	1.2	0.9	1.6	1.6
	Denmark	1.0	0.2	0.2	0.2	0.2	0.1	0.2	0.2
	Estonia	0.9	0.6	0.5	0.4	0.4	0.3	0.6	0.6
	Finland	0.8	1.1	1.0	0.8	0.8	0.6	1.1	1.1
	France	1.1	0.0	0.0	0.0	0.0	0.0	0.0	0.0
	Germany	1.1	0.0	0.0	0.0	0.0	0.0	0.0	0.0
	Greece	0.7	1.4	1.2	1.0	1.0	0.7	1.4	1.4
	Hong Kong SAR, China	1.1	0.0	0.0	0.0	0.0	0.0	0.0	0.0
	Hungary	0.8	1.1	1.0	0.8	0.8	0.6	1.1	1.1
	Iceland	0.9	0.4	0.4	0.3	0.3	0.2	0.4	0.4
	Ireland	1.6	0.0	0.0	0.0	0.0	0.0	0.0	0.0
	Israel	1.1	0.0	0.0	0.0	0.0	0.0	0.0	0.0
	Italy	0.9	0.3	0.3	0.2	0.2	0.2	0.3	0.3
	Korea, Rep.	0.9	0.3	0.3	0.2	0.2	0.2	0.3	0.3
	Kuwait	0.6	2.7	2.3	2.0	2.0	1.4	2.7	2.7
	Latvia	1.0	0.3	0.3	0.2	0.2	0.2	0.3	0.3
	Lithuania	0.9	1.0	0.9	0.7	0.7	0.5	1.0	1.0
	Luxembourg	0.9	0.5	0.4	0.4	0.4	0.3	0.5	0.5
	Malta	1.1	0.0	0.0	0.0	0.0	0.0	0.0	0.0
	Netherlands	0.9	0.4	0.3	0.3	0.3	0.2	0.4	0.4
	New Zealand	0.9	0.7	0.6	0.5	0.5	0.4	0.7	0.7
	Norway	1.0	0.3	0.3	0.2	0.2	0.2	0.3	0.3
	Oman	0.5	4.5	3.9	3.3	3.3	2.4	4.5	4.5
	Panama	0.6	4.9	4.3	3.7	3.6	2.6	4.9	4.9
	Poland	0.8	1.4	1.2	1.0	1.0	0.7	1.4	1.4
	Portugal	0.9	0.3	0.3	0.2	0.2	0.2	0.3	0.3
	Qatar	0.6	2.6	2.2	1.9	1.9	1.4	2.6	2.6
	Saudi Arabia	0.2	7.5	6.5	5.6	5.5	4.0	7.5	7.5
	Seychelles	1.0	0.6	0.5	0.5	0.5	0.3	0.6	0.6
	Slovak Republic	1.0	0.1	0.1	0.1	0.1	0.1	0.1	0.1
	Spain	0.9	0.4	0.4	0.3	0.3	0.2	0.4	0.4
	Sweden	1.0	0.3	0.2	0.2	0.2	0.2	0.3	0.3
	Switzerland	1.0	0.0	0.0	0.0	0.0	0.0	0.0	0.0
	United Arab Emirates	0.8	1.2	1.1	0.9	0.9	0.7	1.2	1.2
	United Kingdom	1.1	0.0	0.0	0.0	0.0	0.0	0.0	0.0
	United States	0.7	2.0	1.8	1.5	1.5	1.1	2.0	2.0
	Uruguay	0.9	0.6	0.5	0.4	0.4	0.3	0.6	0.6
	**High income median**	**0.9**	**0.6**	**0.5**	**0.4**	**0.4**	**0.3**	**0.6**	**0.6**
Upper middle income	Albania	0.8	1.8	1.6	1.3	1.3	0.9	1.8	1.8
	Argentina	0.8	2.4	2.1	1.8	1.8	1.3	2.4	2.4
	Armenia	0.4	5.8	5.0	4.2	4.1	2.9	5.7	5.6
	Azerbaijan	0.4	5.5	4.8	4.0	3.9	2.7	5.5	5.4
	Belarus	1.0	0.1	0.1	0.1	0.1	0.1	0.1	0.1
	Belize	0.5	5.3	4.6	3.8	3.7	2.6	5.3	5.2

Group	Country	2018 Gross Enrollment Rates (%)[a]	COI (% GDP)	Sensitivity analysis					
				i=7	*i=6*	*d=4*	*d=5*	*cost +10%*	*cost +20%*
	Brazil	1.0	0.3	0.3	0.2	0.2	0.2	0.3	0.3
	Bulgaria	0.8	1.5	1.3	1.1	1.1	0.8	1.5	1.5
	China	0.9	1.2	1.0	0.9	0.8	0.6	1.2	1.1
	Costa Rica	1.0	0.2	0.2	0.1	0.1	0.1	0.2	0.2
	Dominican Republic	0.5	6.1	5.3	4.5	4.4	3.2	6.1	6.0
	Ecuador	0.7	3.2	2.8	2.3	2.3	1.6	3.2	3.1
	Grenada	1.0	0.0	0.0	0.0	0.0	0.0	0.0	0.0
	Guatemala	0.5	7.0	6.1	5.1	5.0	3.5	7.0	6.9
	Iran, Islamic Rep.	0.5	4.6	4.0	3.3	3.3	2.3	4.5	4.5
	Jamaica	0.7	2.7	2.3	1.9	1.9	1.4	2.6	2.6
	Jordan	0.3	10.2	8.8	7.4	7.2	5.1	10.1	10.0
	Kazakhstan	0.6	5.5	4.8	4.0	3.9	2.9	5.4	5.4
	Malaysia	1.0	0.1	0.1	0.1	0.1	0.1	0.1	0.1
	Maldives	0.9	1.3	1.1	1.0	0.9	0.7	1.3	1.3
	Mauritius	1.0	0.1	0.1	0.1	0.1	0.1	0.1	0.1
	Mexico	0.7	2.9	2.5	2.1	2.1	1.5	2.9	2.8
	Montenegro	0.7	2.2	1.9	1.6	1.6	1.2	2.2	2.2
	Namibia	0.3	9.6	8.3	7.0	6.8	4.9	9.5	9.4
	North Macedonia	0.4	4.4	3.8	3.3	3.2	2.3	4.4	4.4
	Paraguay	0.4	6.9	6.0	5.1	5.0	3.6	6.9	6.8
	Peru	1.0	0.0	0.0	0.0	0.0	0.0	0.0	0.0
	Romania	0.8	1.1	1.0	0.8	0.8	0.6	1.1	1.1
	Russian Federation	0.9	1.0	0.9	0.8	0.7	0.5	1.0	1.0
	Samoa	0.5	7.8	6.7	5.6	5.5	3.9	7.7	7.6
	Serbia	0.6	3.5	3.0	2.6	2.5	1.8	3.5	3.5
	South Africa	0.2	8.3	7.2	6.1	5.9	4.3	8.3	8.2
	St. Lucia	0.7	1.8	1.5	1.3	1.3	0.9	1.8	1.8
	Suriname	0.9	1.6	1.4	1.2	1.1	0.8	1.6	1.6
	Thailand	0.8	1.5	1.3	1.1	1.1	0.8	1.5	1.5
	Turkey	0.3	7.5	6.5	5.5	5.4	4.0	7.4	7.4
	**Upper middle income median**	**0.7**	**2.5**	**2.2**	**1.9**	**1.8**	**1.3**	**2.5**	**2.5**
Lower middle income	Angola	0.4	9.2	7.9	6.6	6.4	4.5	9.1	9.0
	Bangladesh	0.4	7.8	6.6	5.5	5.4	3.7	7.6	7.5
	Bhutan	0.3	6.9	6.0	5.0	4.9	3.5	6.8	6.8
	Bolivia	0.7	3.8	3.3	2.8	2.7	1.9	3.8	3.7
	Cabo Verde	0.7	3.6	3.1	2.6	2.5	1.8	3.5	3.5
	Cambodia	0.2	10.6	9.0	7.4	7.2	4.8	10.4	10.1
	Cameroon	0.3	10.5	8.8	7.2	7.0	4.5	10.2	9.9
	Comoros	0.2	4.8	3.8	2.8	2.7	1.2	4.5	4.1
	Cote d'Ivoire	0.1	16.1	13.6	11.2	10.9	7.3	15.7	15.4
	Djibouti	0.1	8.3	7.1	5.9	5.7	4.0	8.2	8.0
	Egypt, Arab Rep.	0.3	12.1	10.4	8.7	8.5	6.0	12.0	11.8
	El Salvador	0.7	3.3	2.9	2.4	2.3	1.7	3.3	3.2
	Ghana	1.1	0.0	0.0	0.0	0.0	0.0	0.0	0.0
	Honduras	0.4	6.2	5.3	4.4	4.3	3.0	6.1	6.0
	India	0.1	11.0	9.5	7.9	7.7	5.4	10.9	10.7
	Indonesia	0.6	4.9	4.3	3.6	3.5	2.5	4.9	4.8
	Kenya	0.8	4.6	4.0	3.3	3.2	2.2	4.6	4.5
	Lao PDR	0.5	9.0	7.8	6.5	6.4	4.5	8.9	8.9
	Lesotho	0.4	6.7	5.6	4.5	4.3	2.7	6.5	6.3
	Moldova	0.9	1.1	1.0	0.8	0.8	0.6	1.1	1.1
	Mongolia	0.9	2.2	1.9	1.6	1.6	1.1	2.2	2.1
	Morocco	0.5	6.1	5.3	4.4	4.3	3.1	6.1	6.0
	Papua New Guinea	0.4	6.0	5.1	4.3	4.1	2.8	5.9	5.8
	Philippines	0.8	3.0	2.6	2.2	2.1	1.5	3.0	2.9
	Senegal	0.2	16.0	13.6	11.1	10.8	7.2	15.7	15.3
	Solomon Islands	0.8	2.9	2.5	2.1	2.0	1.4	2.9	2.8
	Sudan	0.5	2.0	1.4	0.7	0.6	-0.4	1.7	1.4
	Timor-Leste	0.2	18.2	15.5	12.8	12.5	8.5	17.9	17.6

Group	Country	2018 Gross Enrollment Rates (%)[a]	COI (% GDP)	Sensitivity analysis					
				i=7	*i=6*	*d=4*	*d=5*	*cost +10%*	*cost +20%*
	Tunisia	0.4	5.0	4.3	3.6	3.5	2.5	5.0	4.9
	Uzbekistan	0.3	10.2	8.7	7.2	7.0	4.7	10.0	9.8
	Vietnam	1.0	0.0	0.0	0.0	0.0	0.0	0.0	0.0
	Zambia	0.1	10.4	8.6	6.8	6.6	3.9	10.0	9.6
	**Lower income median**	**0.4**	**6.2**	**5.3**	**4.4**	**4.3**	**3.0**	**6.1**	**6.0**
Low income	Benin	0.3	8.5	7.0	5.5	5.3	3.0	8.1	7.7
	Burkina Faso	0.0	13.9	11.1	8.3	8.0	3.8	13.1	12.2
	Burundi	0.2	0.0	0.0	0.0	0.0	0.0	0.0	0.0
	Central African Republic	0.0	7.5	5.0	2.6	2.2	-1.4	6.2	5.0
	Chad	0.0	19.3	15.8	12.2	11.8	6.5	18.4	17.5
	Gambia, The	0.4	7.3	5.8	4.2	4.0	1.7	6.8	6.3
	Liberia	1.3	0.0	0.0	0.0	0.0	0.0	0.0	0.0
	Madagascar	0.4	3.5	2.3	1.1	0.9	-0.9	2.9	2.3
	Mali	0.1	14.6	11.9	9.2	8.9	4.9	13.9	13.3
	Nepal	0.9	1.3	1.0	0.8	0.8	0.5	1.2	1.2
	Niger	0.1	12.4	8.8	5.3	4.8	-0.5	10.8	9.2
	Rwanda	0.2	11.1	9.1	7.0	6.7	3.7	10.6	10.0
	Sierra Leone	0.1	6.0	4.1	2.2	2.0	-0.8	5.1	4.2
	Tajikistan	0.1	8.3	6.5	4.7	4.5	1.8	7.7	7.1
	Tanzania	0.4	10.1	8.4	6.7	6.5	4.0	9.8	9.4
	Togo	0.2	9.7	7.7	5.6	5.4	2.4	9.0	8.4
	Uganda	0.1	15.3	12.3	9.3	8.9	4.5	14.5	13.6
	Yemen, Rep.	0.0	10.9	8.8	6.8	6.5	3.4	10.3	9.8
	**Low income median**	**0.1**	**9.1**	**7.3**	**5.4**	**5.0**	**2.1**	**8.6**	**8.1**

[a]Pre-Primary Gross Enrolment Rates in 2018 (2017 if 2018 not available). GER = total enrolments of all ages relative to number of children of pre-primary school age, so it may exceed 100. Source: UNESCO Institute for Statistics (UIS). http://data.uis.unesco.org/

NOTES: pre-primary school attendance is assumed to have an impact on adult earnings of 8%. Children are assumed to enter the labour market at age 18 and benefits are captured during 45 years. Discount rate=3%. Programme costs are assumed to be $1,300, $912, $696 and $654, respectively, for the four country groups. The SDG 4.2 targets are assumed to be enrolment rates equal to the maximum of 100% and the GER for 2018.
