# Supplementary material for: Measuring and forecasting progress in education: what about early childhood?
Source: NPJ Sci Learn. 2021 Sep 10;6:27. doi: 10.1038/s41539-021-00106-7 (PMC8433172; doi:10.1038/s41539-021-00106-7)
Supplement: Supplementary file 2 — Reporting Summary [file 41539_2021_106_MOESM2_ESM.pdf]

nature research

Corresponding author(s): Linda M. Richter

Last updated by author(s): Aug 5, 2021

Reporting Summary

Nature Research wishes to improve the reproducibility of the work that we publish. This form provides structure for consistency and transparency in reporting. For further information on Nature Research policies, see our Editorial Policies and the Editorial Policy Checklist.

Statistics

For all statistical analyses, confirm that the following items are present in the figure legend, table legend, main text, or Methods section.

n/a	Confirmed	
☐	☒	The exact sample size (n) for each experimental group/condition, given as a discrete number and unit of measurement
☐	☒	A statement on whether measurements were taken from distinct samples or whether the same sample was measured repeatedly
☐	☒	The statistical test(s) used AND whether they are one- or two-sided<br>*Only common tests should be described solely by name; describe more complex techniques in the Methods section.*
☐	☒	A description of all covariates tested
☐	☒	A description of any assumptions or corrections, such as tests of normality and adjustment for multiple comparisons
☐	☒	A full description of the statistical parameters including central tendency (e.g. means) or other basic estimates (e.g. regression coefficient) AND variation (e.g. standard deviation) or associated estimates of uncertainty (e.g. confidence intervals)
☐	☒	For null hypothesis testing, the test statistic (e.g. F, t, r) with confidence intervals, effect sizes, degrees of freedom and P value noted<br>*Give P values as exact values whenever suitable.*
☒	☐	For Bayesian analysis, information on the choice of priors and Markov chain Monte Carlo settings
☐	☒	For hierarchical and complex designs, identification of the appropriate level for tests and full reporting of outcomes
☐	☒	Estimates of effect sizes (e.g. Cohen's d, Pearson's r), indicating how they were calculated

Our web collection on statistics for biologists contains articles on many of the points above.

Software and code

Policy information about availability of computer code

Data collection	No primary data collection was carried out for this analysis
Data analysis	All analyses were conducted using R version 3.1.3

For manuscripts utilizing custom algorithms or software that are central to the research but not yet described in published literature, software must be made available to editors and reviewers. We strongly encourage code deposition in a community repository (e.g. GitHub). See the Nature Research guidelines for submitting code & software for further information.

Data

Policy information about availability of data

All manuscripts must include a data availability statement. This statement should provide the following information, where applicable:

- Accession codes, unique identifiers, or web links for publicly available datasets
- A list of figures that have associated raw data
- A description of any restrictions on data availability

This study used data that are available from public online repositories, which require a straightforward registration process and usage agreement with the data provider. Although the authors are restricted from providing the data directly, specific datasets may be made available by request and with permission from the data provider. The authors may be contacted for assistance in acquiring data for the replication of this study. All figures presented in this study have been produced by the authors and no permissions are required for publication.

Field-specific reporting

Please select the one below that is the best fit for your research. If you are not sure, read the appropriate sections before making your selection.

☐ Life sciences ☒ Behavioural & social sciences ☐ Ecological, evolutionary & environmental sciences

For a reference copy of the document with all sections, see nature.com/documents/nr-reporting-summary-flat.pdf

Behavioural & social sciences study design

All studies must disclose on these points even when the disclosure is negative.

Study description	Descriptive, population-level analyses of secondary data on benefits of pre-primary programmes for adolescent mathematics and science scores as well as estimations of the cost of inaction - not making pre-primary education programmes universal - in foregone lifelong earnings.
Research sample	All available nationally representative census and survey data containing information on pre-primary education attendance and educational attainment for:<br>1) A sample of 430,000 children from 73 middle- and high-income countries that were surveyed in the 2018 Programme for International Assessments (PISA).<br>2) A sample of 6.3 billion people in 134 countries with available educational data from UNESCO (2018)
Sampling strategy	No statistical methods were used to predetermine sample size. Cases with variables of interest to the analyses - pre-primary attendance, PISA scores, etc. - were included in the analyses.
Data collection	Not applicable - secondary data analysis
Timing	Not applicable
Data exclusions	Not applicable
Non-participation	Not applicable
Randomization	Not applicable

Reporting for specific materials, systems and methods

We require information from authors about some types of materials, experimental systems and methods used in many studies. Here, indicate whether each material, system or method listed is relevant to your study. If you are not sure if a list item applies to your research, read the appropriate section before selecting a response.

Materials & experimental systems

n/a	Involved in the study	
☒	☐	Antibodies
☒	☐	Eukaryotic cell lines
☒	☐	Palaeontology and archaeology
☒	☐	Animals and other organisms
☒	☐	Human research participants
☒	☐	Clinical data
☒	☐	Dual use research of concern

Methods

n/a	Involved in the study	
☒	☐	ChIP-seq
☒	☐	Flow cytometry
☒	☐	MRI-based neuroimaging
